# Modulation of aqueous humor cytokine profile by faricimab in patients with diabetic macular edema

**DOI:** 10.3389/fmed.2026.1934634

**Published:** 2026-09-10

**Authors:** Zhongqi Wan, Qingquan Wei, Xinxia Chen, Kunhua Jiang, Ying Liu, Qing Peng

**Affiliations:** 1 Department of Ophthalmology, Shanghai Tenth People’s Hospital, School of Medicine, Tongji University, Shanghai, China; 2 Department of Ophthalmology, Shanghai Tenth People’s Hospital Chongming Branch, School of Medicine, Tongji University, Shanghai, China

**Keywords:** angiopoietin-2, biomarkers, diabetic macular edema, faricimab, vascular endothelial growth factor

## Abstract

**Objective:**

To explore how intravitreal faricimab reshapes aqueous humor cytokines in diabetic macular edema (DME), and analyze correlations between cytokine shifts and clinical efficacy.

**Methods:**

This prospective study recruited 10 DME patients and 10 age-matched cataract controls. Aqueous humor was harvested from DME patients at baseline and 12 weeks after three faricimab injections, and from controls during cataract surgery. Multiplex immunoassay measured 12 aqueous cytokines. Clinical indicators included best-corrected visual acuity (BCVA), central macular thickness (CMT) and retinal vessel density detected by spectral-domain OCTA.

**Results:**

Baseline aqueous VEGF, Ang-2, IL-8 and PlGF were markedly higher in DME eyes (all *p* < 0.05). Post-treatment, VEGF and Ang-2 fell below control levels, whereas HGF, IP-10, IL-8 and PlGF further increased (all *p* < 0.05). Longitudinal analysis validated reduced VEGF/Ang-2 and elevated HGF after intervention. The original tight cytokine correlations (Ang-2-VEGF, Spearman’s *ρ* = 0.890, raw *p* = 0.001) were disrupted, while new associations such as HGF-IL-6 (Spearman’s *ρ* = 0.888, raw *p* = 0.001) formed. CMT decreased and BCVA improved significantly (both *p* < 0.05); declines in Ang-2 and VEGF strongly correlated with CMT thinning (Spearman’s *ρ* = 0.824, raw *p* = 0.003; Spearman’s *ρ* = 0.660, raw *p* = 0.034).

**Conclusion:**

Faricimab potently remodels DME aqueous cytokine microenvironment via inhibiting the VEGF/Ang-2 pathway and upregulating HGF, restructuring cytokine crosstalk. There was a significant correlation between reduced target cytokines and improved macular anatomy, which provides preliminary observational evidence supporting dual-pathway inhibition in DME.

## Introduction

Diabetic macular edema (DME), a major vision-threatening complication of diabetic retinopathy, is characterized by fluid accumulation in the macula due to breakdown of the blood-retinal barrier (BRB) (1–4). The pathogenesis of DME is multifactorial and complex, with vascular endothelial growth factor (VEGF) being established as a central mediator, driving vascular hyperpermeability and angiogenesis (5, 6). The clinical success of intravitreal anti-VEGF agents has solidified their role as the first-line therapy for DME (7, 8). However, a significant proportion of patients exhibit suboptimal or non-durable responses to anti-VEGF monotherapy, underscoring the contribution of other non-VEGF pathways in the disease process (7, 9).

Beyond VEGF, inflammatory cascades and the angiopoietin-Tie (Ang-Tie) signaling pathway are increasingly recognized as pivotal players in DME (10–12). Pro-inflammatory cytokines, such as interleukin-6 (IL-6) and IL-8, are markedly elevated in the ocular fluid of DME patients, promoting vascular leakage and leukostasis (13, 14). Concurrently, Angiopoietin-2 (Ang-2), which is upregulated in hypoxic and inflammatory conditions, acts as a context-dependent antagonist of the Tie2 receptor (11, 15). Elevated Ang-2 levels destabilize vasculature, promote inflammation, and synergize with VEGF to exacerbate BRB disruption. This intricate crosstalk between VEGF, Ang-2, and inflammation provides a compelling rationale for dual-pathway inhibition as a potential therapeutic strategy (16–18).

Faricimab is the first intraocular bispecific antibody that simultaneously neutralizes VEGF-A and Ang-2 (16, 17, 19). It is hypothesized that combined blockade of two pathogenic pathways may produce different intraocular cytokine responses compared with single-agent anti-VEGF treatment (20, 21). Clinical trials have demonstrated the efficacy and durability of faricimab in DME (17, 19, 22). However, direct comparative data between faricimab and monotherapy remain insufficient in real-world cohorts. This study only describes cytokine alterations observed after faricimab administration, without comparative data from patients receiving ranibizumab or aflibercept to distinguish drug-specific versus universal anti-VEGF molecular changes.

Therefore, this prospective study aimed to investigate the dynamic changes in the aqueous humor cytokine profile of DME patients following intravitreal faricimab injection. Using a multiplex immunoassay, we quantified the concentrations of a panel of cytokines, including VEGF, Ang-2, and inflammatory factors. Furthermore, we correlated these molecular changes with anatomical biomarkers obtained from optical coherence tomography angiography (OCTA), such as central macular thickness (CMT) and retinal vessel density (23, 24). We hypothesized that faricimab treatment would significantly suppress not only VEGF but also Ang-2 and key inflammatory cytokines, and that these changes would be associated with improved retinal anatomy. Our findings seek to elucidate the molecular underpinnings of faricimab’s efficacy and highlight the potential of aqueous humor biomarkers for monitoring treatment in DME.

## Materials and methods

### Study design and participants

This prospective, observational study enrolled 10 patients with DME and 10 age-matched patients with cataract as controls at the Department of Ophthalmology, Shanghai Tenth People’s Hospital, between March 2024 and March 2025. The study protocol was approved by the Institutional Review Board of Shanghai Tenth People’s Hospital, Tongji University School of Medicine (No. SHSY-IEC-6.0/26K76/P01) and was conducted in accordance with the tenets of the Declaration of Helsinki. Written informed consent was obtained from all participants prior to enrollment. At enrollment, comprehensive systemic and ophthalmic baseline data were collected for all DME subjects to assess potential confounding variables, including diabetes duration, HbA1c, diabetic retinopathy grading, DME disease course, lens status, renal function status, history of prior ocular laser or intraocular surgery, and chronic systemic medication regimens, all of which are summarized in Table 1.

**Table 1:** Demographic characteristics of the patients in the DME and control groups.

Characteristics	DME (*n* = 10)	Cataract (*n* = 10)	*p* value
Participants (*n*)	10	10	-
Age (years)	71.20 ± 5.37	73.80 ± 3.88	0.23
Gender (F/M)	5/5	5/5	1
Eye (Right/Left)	6/4	6/4	1
Diabetes duration	10 ± 1.12	-	-
HbA1c (%)	7.57 ± 0.76	-	-
Diabetic retinopathy severity (*n*, %)	Mild NPDR (2), Moderate NPDR (6), Severe NPDR (2)	-	-
Duration of DME	1.05 ± 0.35	-	-
Lens status	Mild–moderate age-related cataract	Mild–moderate age-related cataract	-
Renal function (*n*, %)	0	0	1
Previous ocular treatment (*n*, %)	0	0	1
Systemic medication use	All received oral hypoglycemic agents; 1 patient on antihypertensives	-	-
Hypertension (*n*, %)	1 (10.0%)	1 (10.0%)	1
Heart disease (*n*, %)	0	0	1

M/W, man/woman; Data represented the mean ± standard deviation (SD) of each group; *p* < 0.05 was considered statistically significant.

### Inclusion and exclusion criteria

Inclusion criteria for the DME group were: (1) diagnosis of type 2 diabetes mellitus with clinically significant macular edema involving the central fovea; (2) best-corrected visual acuity (BCVA) between 20/400 and 20/40 on a Snellen chart; all formal visual acuity measurements at baseline and follow-up were performed with ETDRS charts and converted to LogMAR units for statistical analysis; (3) CMT ≥ 300 μm as measured by spectral-domain optical coherence tomography (SD-OCT); (4) no prior intravitreal anti-VEGF or steroid injection in the study eye within the past 3 months.

Exclusion criteria for the DME group included: (1) presence of macular ischemia, epiretinal membrane, or vitreomacular traction that could confound the assessment of DME; (2) history of panretinal photocoagulation within the past 6 months or any intraocular surgery within the past 3 months in the study eye; (3) any other retinal vascular diseases (e.g., retinal vein occlusion); (4) active ocular infection or inflammation; (5) uncontrolled systemic disease (e.g., hypertension, renal failure); (6) pregnancy or lactation.

The cataract control group consisted of patients undergoing routine phacoemulsification surgery without any other ocular pathologies, particularly diabetic retinopathy or other retinal diseases. All cataract control subjects had no history of type 1 or type 2 diabetes, and their fasting blood glucose and HbA1c levels were within the normal range at enrollment. Subjects with any form of diabetes were excluded from the control group.

### Intervention and sample collection

Patients received a total of three intravitreal faricimab injections on a fixed 4-week schedule: the first injection was given at baseline, with baseline aqueous humor collected immediately before administration; the second injection was performed at Week 4; the third injection was scheduled at Week 12, and follow-up aqueous samples were harvested right prior to this final third injection. No additional fourth injection was administered during the study. All aqueous specimens (≈100 μL) were obtained via limbal paracentesis using a 30-gauge needle and immediately stored at −80 °C after collection. Notably, Week 12 aqueous samples were collected at trough right before the third injection, with a 4-week interval after the second dose. Though this is the cycle’s lowest drug level, trace faricimab remains intraocular, so cytokine changes reflect cumulative prior drug effects instead of a drug-free state.

### Cytokine analysis

The concentrations of 12 cytokines in the aqueous humor were simultaneously quantified using the Bio-Plex Pro Human Cytokine Assay on a Bio-Plex 200 suspension array system (Bio-Rad Laboratories, Inc., USA). The analyzed cytokines included: vascular endothelial growth factor-A (VEGF-A), angiopoietin-2 (Ang-2), platelet-derived growth factor-AA (PDGF-AA), hepatocyte growth factor (HGF), placental growth factor (PlGF), interleukin (IL)-6, IL-8, interferon gamma-induced protein 10 (IP-10), basic fibroblast growth factor (FGF-basic), heparin-binding EGF-like growth factor (HB-EGF), tumor necrosis factor-alpha (TNF-*α*), and IL-1β. All assays were performed in duplicate according to the manufacturer’s instructions, and cytokine concentrations (pg/mL) were determined from standard curves.

### Ophthalmic examinations

All DME patients underwent comprehensive ophthalmic examinations at baseline and at the 12-week follow-up visit. Formal BCVA measurement was performed using Early Treatment Diabetic Retinopathy Study (ETDRS) charts and converted to LogMAR for quantitative analysis; Snellen chart values were only applied during preliminary enrollment screening to judge subject eligibility. Additional examinations included slit-lamp biomicroscopy, intraocular pressure measurement. Optical coherence tomography (OCT) was performed to measure central macular thickness (CMT). Optical coherence tomography angiography (OCTA; Optovue RTVue-XR, USA) was performed using 6×6 mm macular scans to superficial retinal vessel density (SRVD), and deep retinal vessel density (DRVD). OCTA scanning was performed using the Optovue RTVue-XR platform with its original built-in analysis software. All 6 × 6 mm macular scans centered on the fovea were automatically segmented into two capillary plexuses: the superficial plexus spanning from the inner limiting membrane (ILM) to the inner plexiform layer (IPL), and the deep plexus ranging from the inner nuclear layer (INL) to the outer plexiform layer (OPL). All vascular density parameters (superficial foveal vessel density, deep foveal vessel [DFVD]) were calculated as the percentage of vascular pixels within a 1 mm diameter circular zone centered on the fovea. FAZ area (Supplementary Figure 1) and total macular retinal thickness (whole retinal thickness) were automatically generated by the system. Strict image quality control was implemented: only scans with signal strength ≥7/10 were retained; images with motion artifacts, shadow artifacts or media opacities were excluded from statistical analysis. A single experienced grader masked to clinical data reviewed all automatic segmentation contours, and manually corrected misaligned segmentation boundaries before extracting quantitative parameters. Notably, macular cystoid edema often distorts automated segmentation. We defined edema-related errors as shifted or broken layer boundaries caused by intraretinal fluid. For all edematous scans, the masked grader manually realigned ILM, IPL, INL and OPL contours to match native retinal anatomy before calculating vascular parameters; severe edema images underwent double manual review to reduce measurement bias.

### Study outcomes

The primary outcome measures were: (1) Differences in baseline aqueous humor cytokine profiles between DME patients and cataract controls; (2) Longitudinal changes in the concentrations of aqueous humor cytokines (particularly VEGF-A, Ang-2, and inflammatory cytokines) from baseline to Week 12 after faricimab treatment; (3) Changes in anatomical and functional parameters, including BCVA (LogMAR), CMT, SRVD, and DRVD; (4) Correlations between changes in cytokine levels and changes in OCTA parameters. Correlation analyses between cytokines and retinal morphological indicators were performed using absolute measurement values collected at the 12-week follow-up visit.

### Statistical analysis

Statistical analyses were performed using SPSS software (version 23.0; IBM Corp., USA). The normality of all continuous variables was verified via Shapiro–Wilk test and Q-Q plot visualization; all indicators satisfied the normality assumption (all *p* > 0.05). All normally distributed continuous data are presented as mean ± standard deviation (SD). Independent samples *t*-test was used for between-group comparisons (DME vs. cataract control), and paired *t*-test was applied for longitudinal within-group comparisons (baseline vs. Week 12). Spearman’s rank correlation coefficient (*ρ*) was used to assess pairwise relationships between cytokines, as well as correlations between cytokine levels and OCTA parameters; no formal multivariate network modeling was conducted in this study. Accordingly, we only interpret changes in bivariate associations rather than global cytokine network shifts. All correlation coefficients are uniformly labeled Spearman’s *ρ* throughout tables, and the symbol “r” is eliminated. This is a small exploratory pilot study with only 10 DME subjects. All pairwise cytokine correlation analyses were hypothesis-generating exploratory analyses, and multiple comparison correction was not applied to all paired tests. Multiple adjustment methods such as Bonferroni correction would drastically reduce statistical power and increase false-negative risk in this limited sample, masking potentially biologically relevant cytokine interactions. All *p*-values reported in Tables 2, 3 are unadjusted raw two-tailed *p*-values, and correlative findings are preliminary observations requiring validation in larger independent cohorts. A two-tailed *p*-value of less than 0.05 was considered statistically significant. Data visualization was generated using R software (version 4.2.0).

**Table 2:** Cytokine concentration in aqueous humor before and after treatment in DME group and in the cataract group (pg/mL).

Variable	Cataract (*n* = 10)	DME, baseline (pre-treatment) (*n* = 10)	DME, Week 12 (post-treatment) (*n* = 10)	*P*_1_	*P*_2_	*P*_3_
Ang-2	51.11 ± 15.07	87.88 ± 52.81	35.76 ± 15.18	0.048	0.036	0.008
FGF basic	6.74 ± 2.79	7.92 ± 3.85	9.64 ± 4.02	0.445	0.078	0.340
HGF	251.52 ± 91.93	328.85 ± 75.07	467.40 ± 131.81	0.054	<0.001	0.010
IL-6	24.74 ± 20.42	36.01 ± 45.54	30.59 ± 20.08	0.484	0.526	0.735
PDGF-AA	21.83 ± 6.42	28.22 ± 8.41	24.91 ± 7.23	0.072	0.327	0.358
TNF-a	0.76 ± 0.62	0.82 ± 0.64	1.35 ± 2.05	0.817	0.393	0.448
IP-10	40.02 ± 24.07	45.23 ± 15.50	73.20 ± 43.26	0.572	0.047	0.065
HB-EGF	2.66 ± 1.68	0.10 ± 2.56	3.42 ± 1.69	0.827	0.323	0.326
IL-1b	5.30 ± 4.99	4.29 ± 5.30	6.84 ± 6.30	0.668	0.550	0.340
IL8	10.77 ± 6.53	27.71 ± 15.91	35.95 ± 20.88	0.006	0.002	0.334
PlGF	0.71 ± 0.28	2.90 ± 2.60	1.38 ± 0.43	0.016	0.001	0.084
VEGF	100.96 ± 39.66	571.54 ± 667.84	2.81 ± 1.93	0.039	<0.001	0.015

P_1_, DME, baseline (pre-treatment) vs. cataract; P_2_, DME, Week 12 (post-treatment) vs. cataract; P3, DME, Week 12 (post-treatment) vs. DME, baseline (pre-treatment). Paired *t*-tests were adopted for all P3 intra-group longitudinal comparisons of the same DME subjects.

**Table 3:** Pairwise Spearman correlations of aqueous cytokines in DME patients at baseline.

Variable	Ang-2	FGF basic	HGF	IL-6	PDGF-AA	TNF-a	IP-10	HB-EGF	IL-1b	IL-8	PIGF	VEGF
	Spearman’s *ρ*	Spearman’s *ρ*	Spearman’s *ρ*	Spearman’s *ρ*	Spearman’s *ρ*	Spearman’s *ρ*	Spearman’s *ρ*	Spearman’s *ρ*	Spearman’s *ρ*	Spearman’s *ρ*	Spearman’s *ρ*	Spearman’s *ρ*
	*p* value	*p* value	*p* value	*p* value	*p* value	*p* value	*p* value	*p* value	*p* value	*p* value	*p* value	*p* value
Ang-2		0.385	−0.101	−0.128	−0.414	−0.286	0.720	−0.223	−0.291	0.768	0.432	0.890
		0.272	0.782	0.724	0.234	0.424	0.019	0.536	0.414	0.009	0.212	0.001
FGF basic			−0.492	0.238	−0.124	−0.047	0.525	0.351	0.366	−0.232	0.077	0.500
			0.148	0.507	0.732	0.897	0.119	0.320	0.298	0.445	−0.075	0.141
HGF				0.149	0.636	0.183	−0.067	−0.330	−0.388	−0.291	−0.105	−0.249
				0.680	0.048	0.613	0.854	0.351	0.267	0.414	0.774	0.487
IL-6					0.229	−0.245	0.146	−0.046	0.087	0.474	−0.172	0.122
					0.524	0.496	0.688	0.900	0.812	0.166	0.634	0.738
PDGF-AA						0.270	−0.463	0.209	−0.550	−0.244	−0.278	−0.421
						0.450	0.177	0.563	0.100	0.497	0.437	0.226
TNF-a							−0.278	0.000	−0.060	−0.488	0.231	−0.401
							0.437	1.000	0.870	0.153	0.520	0.250
IP-10								−0.177	−0.031	0.722	0.067	0.721
								0.625	0.933	0.612	0.853	0.019
HB-EGF									0.385	−0.159	−0.623	−0.128
									0.272	0.662	0.054	0.724
IL-1b										−0.216	−0.206	−0.167
										0.549	0.568	0.645
IL-8											0.276	0.903
											0.440	0.000
PIGF												0.411
												0.238

All correlations are reported as Spearman’s *ρ*. Only unadjusted raw two-tailed *p* values are displayed; no multiple comparison correction was applied for all pairwise cytokine analyses. This small exploratory cohort (*n* = 10 DME eyes) cannot provide definitive causal conclusions, and all correlative trends require validation in larger independent patient groups.

## Results

### Study population and baseline characteristics

This study enrolled 10 patients with DME (10 eyes), comprising 5 males and 5 females, with a mean age of (71.20 ± 5.37) years. Among these, 6 were right eyes and 4 were left eyes. Additionally, the cataract control group included 10 patients (10 eyes), consisting of 5 males and 5 females, with a mean age of (73.80 ± 3.88) years, including 6 right eyes and 4 left eyes. There were no statistically significant differences between the DME group and the cataract control group in terms of age, gender distribution, or ocular distribution. Furthermore, no statistically significant differences were observed in the prevalence of systemic diseases such as heart disease or hypertension between the DME group and the control group (Table 1). For DME patients, the mean diabetes duration was 10 ± 1.12 years, with an average HbA1c of 7.57 ± 0.76%. All subjects presented non-proliferative diabetic retinopathy: 2 mild NPDR, 6 moderate NPDR, and 2 severe NPDR cases. The mean duration of clinically significant macular edema was 1.05 ± 0.35 years. All patients had normal renal function at baseline. Three participants received panretinal photocoagulation more than 6 months before enrollment, and none underwent intraocular injections or intraocular surgery within 3 months prior to study entry. Systemic medication profiles showed all patients received oral hypoglycemic agents, one patient took long-term antihypertensive drugs. All study eyes with DME had mild-to-moderate age-related cataracts. The degree of lens opacity did not impede OCTA and fundus examinations, and none of the participants had undergone previous cataract surgery. No severe lens sclerosis or dense nuclear cataract was observed in any DME subject.

### Alterations in the aqueous humor cytokine profile

Prior to the initial injection, the concentrations of VEGF, IL-8, PlGF, and Ang-2 in the aqueous humor of DME-affected eyes were significantly higher than those in the cataract control group (all *p* < 0.05). In contrast, no statistically significant differences were observed in the baseline levels of HGF, PDGF-AA, IP-10, FGF-basic, HB-EGF, TNF-*α*, IL-1β, and IL-6 between the two groups (all *p* > 0.05).

Following intravitreal faricimab treatment, the concentrations of VEGF and Ang-2 in the aqueous humor of the DME group were significantly lower than those in the cataract control group (both *p* < 0.05), while the concentrations of HGF, IP-10, IL-8, and PlGF were further significantly elevated compared with the cataract control group (all *p* < 0.05). In contrast, there were no statistically significant differences in the levels of PDGF-AA, FGF-basic, HB-EGF, TNF-*α*, IL-1β, and IL-6 between the two groups after treatment (all *p* > 0.05).

Further longitudinal analysis of the DME group revealed that intravitreal faricimab injection significantly altered the aqueous humor cytokine profile: VEGF and Ang-2 were significantly decreased after treatment, while the concentration of HGF was further significantly increased compared with the pre-treatment level (*p* < 0.05). No significant changes were detected in the levels of PDGF-AA, FGF-basic, HB-EGF, IP-10, TNF-*α*, IL-1β, IL-6, IL-8, and PlGF (all *p* > 0.05) (Table 2).

### Correlations between cytokines

Exploratory pairwise correlation analysis preliminarily detected several positive associations among cytokines in DME eyes at baseline without multiple comparison adjustment: Ang-2 was correlated with VEGF (Spearman’s *ρ* = 0.890, raw *p* = 0.001), IP-10 (Spearman’s *ρ* = 0.720, raw *p* = 0.019), and IL-8 (Spearman’s *ρ* = 0.768, raw *p* = 0.009); HGF was correlated with PDGF-AA (Spearman’s *ρ* = 0.636, raw *p* = 0.048); IP-10 was correlated with VEGF (Spearman’s *ρ* = 0.721, raw *p* = 0.019); and IL-8 was correlated with VEGF (Spearman’s *ρ* = 0.903, raw *p* < 0.001) (Table 2). These associations represent preliminary exploratory findings from a small sample and need further verification in large cohorts (Table 3).

Post-treatment cytokine correlative patterns were substantially remodeled in exploratory unadjusted analyses: Ang-2 was positively correlated with IP-10 (Spearman’s *ρ* = 0.724, raw *p* = 0.018); HGF exhibited a strong positive correlation with IL-6 (Spearman’s *ρ* = 0.888, raw *p* = 0.001) (Table 3). No multiple comparison correction was performed, so these correlative trends cannot be regarded as definitive conclusions (Table 4).

**Table 4:** Pairwise Spearman correlations of aqueous cytokines in DME patients at week 12 after faricimab therapy.

Variable	Ang-2	FGF basic	HGF	IL-6	PDGF-AA	TNF-a	IP-10	HB-EGF	IL-1b	IL-8	PIGF	VEGF
	Spearman’s *ρ*	Spearman’s *ρ*	Spearman’s *ρ*	Spearman’s *ρ*	Spearman’s *ρ*	Spearman’s *ρ*	Spearman’s *ρ*	Spearman’s *ρ*	Spearman’s *ρ*	Spearman’s *ρ*	Spearman’s *ρ*	Spearman’s *ρ*
	*p* value	*p* value	*p* value	*p* value	*p* value	*p* value	*p* value	*p* value	*p* value	*p* value	*p* value	*p* value
Ang-2		−0.346	0.107	0.268	0.346	0.107	0.724	0.108	0.114	0.249	−0.095	0.381
		0.327	0.769	0.454	0.327	0.769	0.018	0.767	0.755	0.487	0.794	0.278
FGF basic			−0.318	−0.055	−0.183	0.038	−0.373	−0.282	−0.331	−0.052	0.529	0.045
			0.370	0.880	0.612	0.918	0.288	0.431	0.350	0.886	0.116	0.902
HGF				0.888	0.418	−0.362	0.200	0.214	−0.375	0.286	0.377	0.272
				0.001	0.229	0.305	0.580	0.553	0.285	0.424	−0.232	0.209
IL-6					0.310	−0.506	0.255	0.257	−0.580	0.338	0.021	−0.158
					0.383	0.135	0.476	0.473	0.079	0.339	0.953	0.663
PDGF-AA						0.362	0.394	−0.376	−0.250	0.316	0.244	0.589
						0.305	0.260	0.284	0.486	0.374	0.497	0.073
TNF-a							0.106	−0.527	0.386	−0.156	0.110	0.511
							0.771	0.117	0.271	0.666	0.763	0.131
IP-10								−0.143	−0.063	0.365	−0.213	0.562
								0.694	0.864	0.300	0.554	0.091
HB-EGF									0.251	0.508	−0.255	−0.558
									0.481	0.134	0.478	0.356
IL-1b										−0.125	−0.425	0.035
										0.730	0.221	0.923
IL-8											−0.018	0.213
											0.960	0.554
PIGF												−0.176
												0.627

All correlations are reported as Spearman’s *ρ*. Only unadjusted raw two-tailed *p* values are displayed; no multiple comparison correction was applied for all pairwise cytokine analyses. This small exploratory cohort (*n* = 10 DME eyes) cannot provide definitive causal conclusions, and all correlative trends require validation in larger independent patient groups.

### Improvements in anatomical and functional parameters

**Figure 1:**
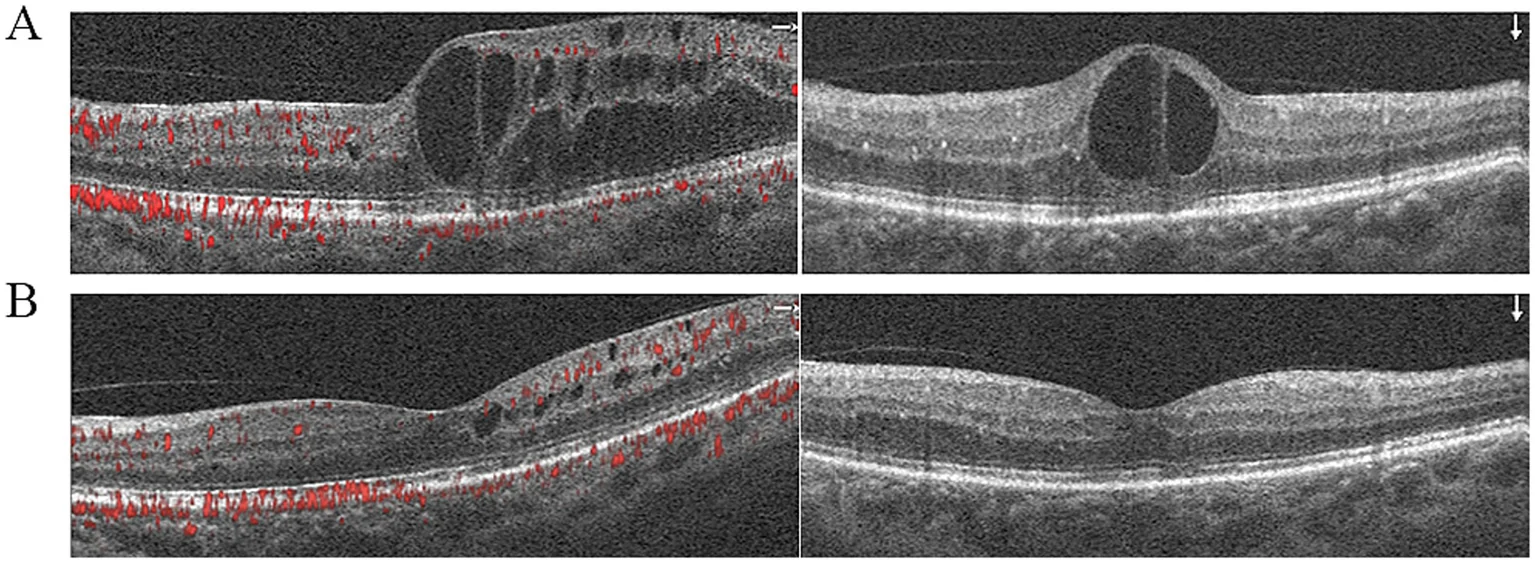
Representative OCT B-scans with superimposed vascular flow signals obtained from a patient with diabetic macular edema (DME). **(A)** Baseline scan acquired before the first intravitreal faricimab injection. **(B)** Corresponding OCT B-scan at the 12-week follow-up. OCT, optical coherence tomography.

OCTA demonstrated significant anatomical improvement following faricimab treatment CMT was significantly reduced (*p* < 0.05). However, no significant changes were observed in full retinal thickness or vessel density parameters—including SRVD, DRVD, superficial foveal vessel density, deep foveal vessel density, or FAZ metrics (all *p* > 0.05). BCVA showed significant improvement (*p* < 0.05) (Table 5). Figure 1 illustrates the response of a typical patient with DME to intravitreal faricimab therapy, showing changes in OCTA.

**Table 5:** Comparison of ophthalmic parameters at baseline and Week 12 in patients with DME.

Variable	DME, baseline (pre-treatment) (*n* = 10)	DME, Week 12 (post-treatment) (*n* = 10)	*p* value
BCVA (logMAR)	0.94 ± 0.45	0.30 ± 0.25	0.002
SRVD	43.92 ± 2.41	44.10 ± 4.39	0.911
DRVD	44.40 ± 2.07	41.96 ± 5.54	0.209
Superficial foveal vessel density	20.54 ± 4.92	18.18 ± 6.78	0.385
Deep foveal vessel density	36.32 ± 12.01	29.28 ± 13.89	0.241
DFVD	44.88 ± 7.95	45.83 ± 6.96	0.779
FAZ	0.21 ± 0.12	0.56 ± 0.68	0.132
Whole retinal thickness (μm)	353.20 ± 38.80	335.6 ± 32.40	0.285
CMT (μm)	368.40 ± 69.25	286.1 ± 57.11	0.010

BCVA, best corrected visual acuity; SRVD, superficial retinal vessel density; DRVD, deep retinal vessel density; DFVD, deep foveal vessel density; CMT, central macular thickness.

### Correlations between cytokines and OCT/OCTA parameters

**Figure 2:**
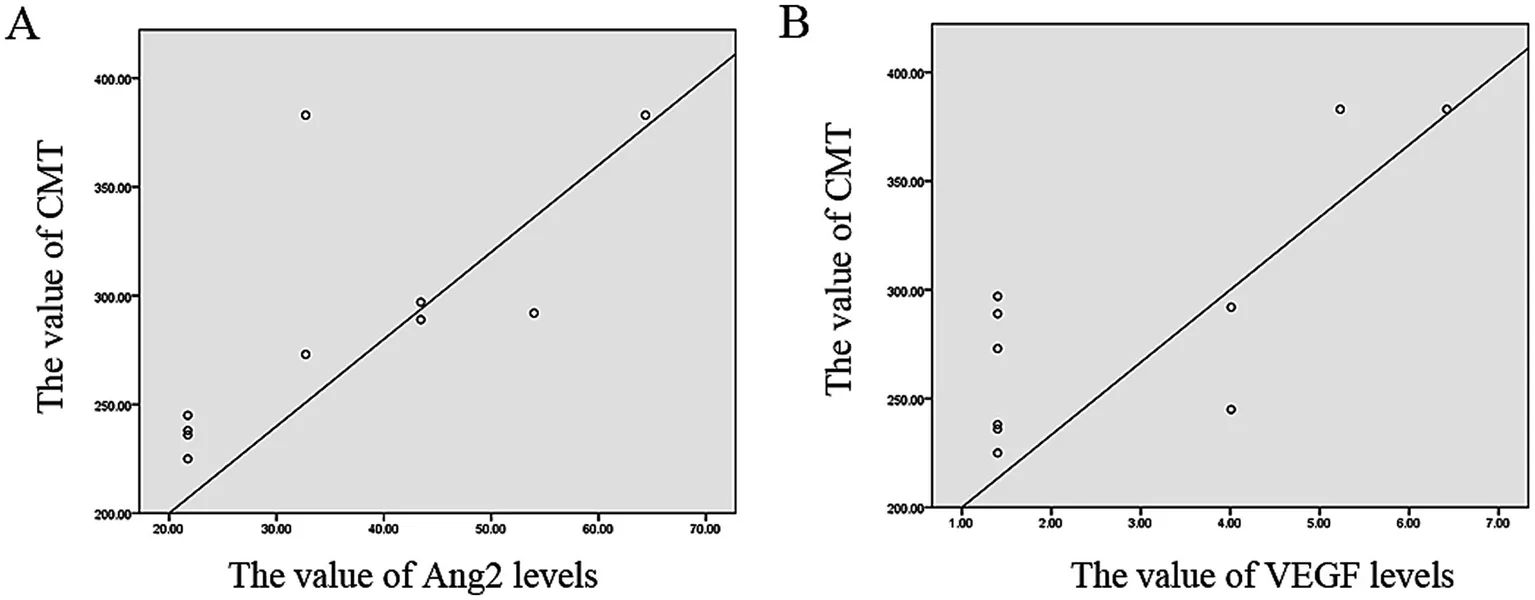
Correlations between cytokine levels and CMT at Week 12. **(A)** Significant positive correlation between Ang-2 level and CMT. **(B)** Significant positive correlation between VEGF level and CMT.

Correlation analysis between cytokine levels and OCT parameters revealed that the reduction in Ang2 levels was positively correlated with the decrease in CMT (Spearman’s *ρ* = 0.824, raw *p* = 0.003), and the reduction in VEGF levels was also positively correlated with the decrease in CMT (Spearman’s *ρ* = 0.660, raw *p* = 0.0338) (Figure 2).

## Discussion

This prospective study systematically elucidates the effects of intravitreal faricimab injection on the aqueous humor cytokine profile and clinical outcomes in patients with DME. The key findings include: (1) Faricimab effectively suppressed its primary targets, VEGF and Ang-2, while inducing a significant increase in HGF levels; (2) The pairwise correlative relationships between cytokines were altered after treatment, disrupting critical pathological associations present in the diseased state; (3) The reduction in key targeted cytokines (VEGF, Ang-2) showed strong positive correlations with the decrease in central macular thickness and coincided with improvements in best-corrected visual acuity. Collectively, these correlative observations describe intraocular cytokine shifts following faricimab monotherapy in a small DME cohort. Without a parallel anti-VEGF monotherapy control group, we cannot confirm whether these molecular alterations are unique to dual VEGF/Ang-2 blockade or represent shared responses to VEGF suppression alone.

In this study, the significantly elevated concentrations of VEGF, Ang-2, IL-8, and PlGF in the aqueous humor of DME-affected eyes compared to controls before treatment align with the pathophysiology of DME as a multifactorial disease involving vascular hyperpermeability, inflammation, and vascular instability (3, 25, 26). Notably, all cataract controls were non-diabetic individuals without elevated glycemic markers. Therefore, the significantly altered cytokine concentrations observed in DME eyes at baseline are specific to macular edema lesions, rather than general systemic inflammation induced by diabetes. It should be emphasized that all cytokine pairwise correlations in this study were derived from unadjusted exploratory analyses in a limited sample of 10 patients without multiple comparison correction. The correlative crosstalk patterns described below are preliminary observational trends rather than confirmed stable intraocular regulatory relationships. Of particular importance, the extremely strong positive correlation between Ang-2 and VEGF (Spearman’s *ρ* = 0.890, raw *p* = 0.001) before treatment, along with the close associations of IP-10 and IL-8 with both, reveals a highly synergistic “vascular-inflammatory” core network. Ang-2, as a vascular destabilizing factor, can enhance endothelial sensitivity to VEGF and promote inflammation (27). The tight coupling of this pathological cytokine network may partially explain suboptimal responses to single anti-VEGF agents, which supports the theoretical value of dual-pathway intervention. Nevertheless, we lack comparative data to verify whether the cytokine remodeling seen in our cohort only occurs under dual inhibition.

Post-treatment, the most significant changes were the marked reduction in VEGF and Ang-2 concentrations compared to the control group and the dissolution of the strong pre-treatment correlation between them. We observed decoupling of pathological cytokine correlations after faricimab treatment. However, without a ranibizumab/aflibercept control group, we cannot attribute this cytokine network rearrangement specifically to concurrent VEGF/Ang-2 inhibition; identical cytokine shifts may also occur following single anti-VEGF therapy (8, 19). The synergistic inhibition of the VEGF/Ang-2 axis may produce a more potent and durable anti-edema effect than single-pathway inhibition by stabilizing vascular endothelium and reducing leakage (8, 28–31). This “decoupling” effect is key to understanding its clinical advantage. Concurrently, the strong positive correlation between the reduction in Ang-2 levels and the decrease in CMT (*r* = 0.824), and the significant correlation between VEGF reduction and CMT improvement (*r* = 0.660), we observed a strong positive correlation between the magnitude of VEGF/Ang-2 decline and the degree of CMT reduction, which only reflects parallel changes rather than a causal relationship. Notably, this strong positive association merely reflects parallel synchronous alterations of two indicators in this observational small cohort and does not confirm a causal relationship. Multiple intraocular confounding factors, including sustained inflammatory microenvironment, may simultaneously modulate cytokine expression and macular fluid retention.

Another notable observational finding of this small cohort was the significant elevation of aqueous HGF at Week 12, alongside a robust positive correlation between post-treatment HGF and IL-6 (*ρ* = 0.888). HGF is a pleiotropic factor with reported vascular protective, anti-apoptotic and tissue-repairing bioactivities (32). Based on this correlative result, we tentatively hypothesize that HGF upregulation might reflect endogenous homeostatic or reparative remodeling after dual VEGF/Ang-2 suppression. Importantly, this mechanistic inference cannot be validated solely from our observational data, and multiple alternative interpretations should be considered: (1) Compensatory cytokine feedback: Suppression of VEGF/Ang-2 signaling may trigger a compensatory upregulation of HGF as a natural counterbalance to vascular leakage; (2) Residual intraocular inflammatory activity: Sustained low-grade ocular inflammation after injection could non-specifically stimulate HGF secretion independent of tissue repair; (3) Random statistical fluctuation: The small sample size (*n* = 10 DME eyes) increases the risk of chance cytokine shifts that do not reflect consistent biological regulation. The tight correlation between HGF and IL-6 further complicates interpretation, as IL-6 exerts dual pro-inflammatory and regenerative effects (33). Accordingly, we cannot definitively conclude that HGF elevation indicates a shift toward reparative intraocular homeostasis; this preliminary association merely provides a testable hypothesis requiring further verification.

Regarding clinical outcomes, the significant reduction in CMT and improvement in BCVA confirm the treatment’s effectiveness, which aligns with the conclusions of multiple Phase III clinical trials and real-world studies (28, 34–39). Notably, no significant changes were observed in retinal vessel density parameters measured by OCTA, such as superficial retinal vessel density SRVD and DRVD, during the short observation period of this study. Previous research suggests that changes in retinal vessel density often lag behind the resolution of macular edema, typically requiring 6 to 12 months of continuous treatment to observe significant optimization of vascular morphology (40). This may explain the lack of significant vascular density changes in our short-term follow-up. This finding likely reflects that the treatment primarily acts on vascular permeability rather than causing immediate significant regression of capillary morphology. Stable vessel density, combined with resolved edema and improved vision, may represent a positive signal, indicating maintained retinal perfusion alongside reduced leakage.

This study has several limitations. First, the small sample size may affect statistical power and the generalizability of the conclusions. Second, the absence of an active control group receiving single-agent anti-VEGF therapy restricts our ability to differentiate whether the detected cytokine alterations stem specifically from Ang-2 co-inhibition, or merely represent shared molecular consequences following VEGF suppression alone. Head-to-head comparative studies with standard anti-VEGF monotherapy arms are required to disentangle dual-target-specific responses from general VEGF-related intraocular cytokine shifts. Third, the 12-week follow-up period is insufficient to assess the long-term dynamics of these molecular changes and their relationship with treatment durability. Fourth, aqueous humor samples reflect the anterior segment microenvironment, which may differ from the local pathological changes in the retina. Fifth, without single anti-VEGF controls, we cannot confirm if the observed cytokine shifts are dual-pathway-specific. Comparative trials are needed to clarify this point. In addition, all mechanistic speculations regarding elevated HGF remain unconfirmed within the present limited dataset. Larger independent longitudinal patient cohorts are required to validate whether HGF upregulation truly represents a consistent homeostatic or reparative response following dual VEGF/Ang-2 pathway inhibition. Additionally, Week 12 samples were collected at trough. Residual faricimab in the eye means cytokine shifts carry cumulative drug effects, a limitation of our sampling design.

## Conclusion

In this small exploratory cohort of DME patients receiving faricimab, we observed suppressed VEGF/Ang-2, elevated HGF and rearranged cytokine interrelations accompanied by improved macular structure. However, correlation data cannot confirm causal links between cytokine changes and anatomical recovery, and the absence of a single anti-VEGF comparison group prevents us from distinguishing these molecular shifts as dual-pathway-specific phenomena. All mechanistic inferences including potential HGF-related homeostatic responses remain unvalidated and require larger controlled studies for verification.

## Data Availability

The original contributions presented in the study are included in the article/supplementary material, further inquiries can be directed to the corresponding authors.
